# Building capacity without disrupting health services: public health education for Africa through distance learning

**DOI:** 10.1186/1478-4491-7-28

**Published:** 2009-04-01

**Authors:** Lucy Alexander, Ehi Uche Igumbor, David Sanders

**Affiliations:** 1 School of Public Health, University of the Western Cape, Bellville, South Africa

## Abstract

The human resources crisis in Africa is especially acute in the public health field. Through distance education, the School of Public Health of the University of the Western Cape, South Africa, has provided access to master's level public health education for health professionals from more than 20 African countries while they remain in post. Since 2000, interest has increased overwhelmingly to a point where four times more applications are received than can be accommodated. This home-grown programme remains sensitive to the needs of the target learners while engaging them in high-quality learning applied in their own work contexts.

This brief paper describes the innovative aspects of the programme, offering some evaluative indications of its impact, and reviews how the delivery of text-led distance learning has facilitated the realization of the objectives of public health training. Strategies are proposed for scaling up such a programme to meet the growing need in this essential area of health human resource capacity development in Africa.

## Review

The human resources crisis in Africa is especially acute in the public health field. Sadana and Petrakova [1] note the concentration of public health programmes in "high-income countries" while IJsselmuiden et al. [2] draw attention to the insufficient number of public health programmes in Africa and their limited coverage arising from their inadequate staffing allocation, among other factors.

In 1993, when the University of the Western Cape (UWC) established its Public Health Programme (which became a School of Public Health in 2000), public health education in South Africa was concentrated in university medical faculties and did not cater for the broad range of allied health professionals working in the health services. Recognizing the need for "... an adequate supply of equitably distributed and competent personnel" [3], to address the country's public health challenges, the UWC undertook to:

• provide an academic environment for appropriate education and training, research and service-oriented courses in the field of public health;

• provide field training that is community-based and fosters community partnership;

• create a centre for innovative ideas in public health education and research, and become a magnet for international health scholars;

• provide a forum for discussion and debate about ethical issues in public health, and empower communities to participate in these debates;

• cooperate with future schools of public health in South Africa, the African continent and internationally [Unpublished document: University of the Western Cape: *Colloquium: The Development of a Western Cape School of Public Health*. Cape Town; 3 February 1992].

In this paper we describe the development of the public health training programme at UWC, emphasizing the innovative aspects of the programme and offering some evaluative indications of its impact. We further demonstrate how, by using the text-led distance learning mode, the School of Public Health (SOPH) has realized the objectives of its training programme. Strategies are proposed for scaling up such a programme to meet the growing need in this essential area of health human resource capacity development in Africa.

## Initiating public health education at UWC

The nascent PHP initially took the form of non-formal summer school and winter school programmes providing continuing professional development courses to a wide range of nurses and mid-level health services managers from South Africa and southern Africa. Thirty such events have taken place to date and through them, more than 7000 health and allied workers have been exposed to the latest thinking in public health, enabling them to critically review planning and implementation of primary health care (PHC) in the health services.

Most such courses last a week, allowing busy health professionals to attend with minimal disruption of the services. The gauge of their success has been the large numbers of health workers they continue to attract, and the fact that many participants return to take further courses. In the early years, the PHP also used these short courses as the contact teaching for the new M Phil in Public Health. In the conversion of this programme to a predominantly distance learning format, these courses have become (optional) contact sessions for the formal postgraduate programme in public health.

A concern to avoid disrupting the health services has driven the conceptualization of the formal programme, which was first offered in 1994 at a time when there was no dedicated and universally accessible public health education in South Africa. A relatively small number of students – 65 – registered over the six years prior to 2000 and by the end of that period, only 19 (33%) of those who could have graduated (N = 57) had done so. The low numbers of registered students, slow throughput of graduates and their consequent per capita cost to the institution over this protracted period was a source of concern, as was the SOPH's low staff complement. In addition, half the students were able to afford their studies and to attend mandatory three-week contact sessions only through external bursary support, which was terminated by the end of 2001.

In the late 1990s, the need to reach more students and to offer training with greater flexibility was identified:

"New strategies have to be found to not only bring training opportunities to health workers, but also to train them while in post, using their own work situation as the practical arena in which to implement the theoretical concepts mastered" [Unpublished document. Sanders D: *Education and Training in Public Health*. UWC: PHP; 2000].

Such access implied financial affordability to mature health professionals with adult responsibilities, as well as opportunities for study in relation to time and place. The target group included a wide range of health, welfare and even education personnel who were full-time employees at district or facility level, located across South Africa, employed in the resource-constrained public sector or in nongovernmental organizations. They were likely to be mature adults with responsibilities for family and career with limited funds for their own study and travel. In South Africa they were also studying in a context of limited access to bursaries or employer support, and therefore little or no possibility of full-time study and limited opportunities to leave the workplace for study purposes. A model of distance education with minimal demands for face-to-face attendance was therefore a logical choice, enabling the school to reach increasing numbers of health workers at lower institutional costs while enabling them to continue to earn a living.

Also important was, as far as possible, to offer an open-learning system allowing students to proceed at their own pace, according to time available. These advantages have been significant, but equally important is the cost saving achieved by retaining the services of trained staff in the health sector for three to four years while they study. In the context of the brain drain to developed countries and dwindling human resources in the health services, this was an important consideration. In the subsequent eight years (2000–2007), the mode of study plus increased staff numbers enabled SOPH to expand its registration by a further 541 students, increasing the average annual number of new students from 10 in the first six years to 67 students per annum thereafter.

**Figure 1:**
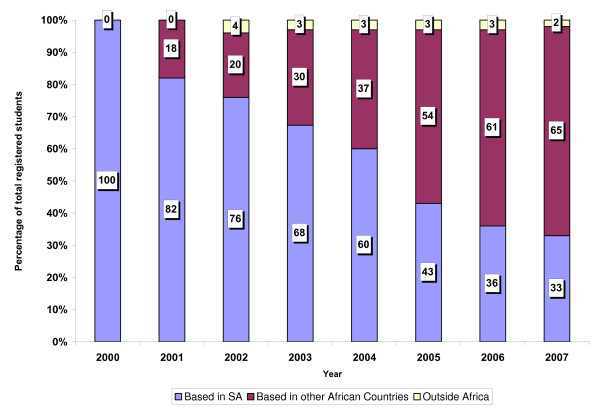
**Country of residence of registered students in percentages by year**.

The student profile has also changed over the past 13 years [Figure 1], from an almost 100% South African group through 2000 to a multi-nation African student community based in more than 20 African countries, including Botswana, Cameroon, Djibouti, Ethiopia, Ghana, Kenya, Lesotho, Malawi, Mozambique, Namibia, Niger, Nigeria, Rwanda, Tanzania, Senegal, South Africa, Sudan, Swaziland, Uganda, Zambia and Zimbabwe. By 2007, 33% of the students were in South Africa, 65% from other African countries and a small number outside the African continent.

In addition to offering the potential to upscale and broaden delivery of the programme, the choice of a predominantly distance mode of study has had the following institutional and pedagogical advantages:

• Reducing lecturing staff numbers makes possible reduced institutional costs, i.e. a small number of subject experts can write material for large numbers of students, while senior students and new graduates can tutor, assess and support students.

• Studying while working facilitates the integration of new conceptual knowledge with practice through the application of problems to real situations in assignments and projects.

• After an initial period of adaptation, studying at a distance nurtures self-directed learning habits and meets the needs of those who are already more self-directed, allowing them a choice of pace and approach.

## Innovative aspects of the programme, 2000–2007

Within the postgraduate programme, innovation has been responsive to student needs in many ways. Although no formal needs analysis was undertaken at the outset, the experience of training South African health professionals over the previous six years provided invaluable knowledge of the working lives, learning needs and priorities of the health professionals for whom the programme was designed. Developing a programme that is responsive to students' needs [4] may not generally be regarded as innovative, but is a necessary corrective to teacher- or institution-centredness in programme design and has resonance with what Young terms a "connective-curriculum design ...which not only shapes learner purpose but is shaped by them" [5]. This orientation has been important in the programme's impact and has been sustained by a series of ongoing internal monitoring and evaluation processes.

On the basis of review, the M Phil in Public Health was expanded, restructured and renamed in 2000. The most influential change was reshaping the programme into three tiers: the postgraduate certificate in public health (the first step of postgraduate study); postgraduate diploma in public health (comprising the coursework component of the MPH); and a Master's in Public Health (MPH). Those with bachelor's-level qualifications (many of them nurses) entered at postgraduate certificate level, taking a course that was in essence a response to the role change demanded by the district health system.

In South Africa, many nurses were for the first time appointed to management roles in district, regional or even provincial settings, or to programme-specific management roles, e.g. nutrition managers. In this course, they were exposed to a core curriculum covering a wide range of public health topics relevant to a developing country context, issues and current debates in primary health care, public health, heath management and health promotion strategies, basic epidemiological skills and an introduction to research skills.

The concept of innovation suggests newness, alternative learning media and methods, and new technology. However, it has been necessary for the SOPH to restrain its use of technology and media innovations amidst strong peer and institutional urging to switch to web-based learning [6,7]. Though this medium may have the potential to render the institution's task easier, SOPH's learners and their learning environments are not yet ready for e-learning, in that surveys conducted indicate that fewer than a third of our students have prolonged, reliable, high-quality Internet access when they need it.

The web remains a valuable tool for information retrieval, communication and support for our programme, however, and the potential to expand its usage has been regularly tested and monitored and will be more fully developed when the context allows. So far we have been able to offer two elective options for those who have such access, and tested the use of a web-based discussion forum with disappointing results.

Another recognized need was for a qualification for those with a four-year professional qualification who required in-depth coverage of public health topics without embarking on an extended research project. The postgraduate diploma in public health was designed to provide a qualification with some level of specialization in one of six public health streams in accordance with their professional needs, inter alia human resources management, health promotion, health management, health information systems and nutrition management. Finally, students could complete the requirements of the postgraduate diploma as coursework for the MPH, and proceed to complete the master's with a 7500 to 20 000 word mini-thesis.

In 2000, a "mixed-mode" of study comprising predominantly distance learning with mandatory three-week block contact sessions twice a year was replaced with optional attendance of summer and winter school contact sessions. The key shift was to make home-developed text-based module guides the lead medium for learning, of which the SOPH now offers a selection of 22, plus one CD-based module and one e-learning module.

From the outset, the curriculum was conceptualized to accommodate a multidisciplinary group of professionals with health, education and welfare backgrounds, and to contribute to transforming the post-apartheid South African health system in its massive reorientation towards a decentralized district health system underpinned by a PHC approach. Pursuant to achieving this ideal, the curriculum covered themes such as health measurement, health research, quantitative and qualitative methods, health systems and information management, health promotion, health human resources, maternal and child health and nutrition, and the epidemiology of communicable and noncommunicable diseases.

Each module is made up of study sessions written as far as possible in the voice of a peer; these materials were designed using the "guided didactic conversation" approach [8]. In the course of these written study sessions, students encounter regular integrated topical and academic questions, problem-solving tasks and activities, and a strong demand to read widely, with topics cross-referenced to relevant literature that is provided in the form of reading compilations. The materials make demands on students to integrate and apply new concepts, models, strategies and approaches to common practical problems frequently encountered by managers and practitioners in the health services. Aside from the pressure of combining work and study, students gain considerably from this pedagogical model, which facilitates the immediate application of theoretical concepts and models to their situations in the work arena [3].

A wide range of professional experience among staff was an essential component in the process of developing these modules, which were based on the week-long interactive short courses taught over several years and their accompanying files. In consequence, intended outcomes, learning paths, reading selections and activities had in part been mapped out, and the short course experience was, as far as possible, translated into text.

To substitute for the loss of face-to-face contact time for some students, increased emphasis was placed on integrated forms of student support such as problem-solving tasks in the text and assignments, regular e-mail feedback from lecturers on assignments and their drafts, as well as support by telephone where possible. Furthermore, mini-thesis students are encouraged to take leave and spend up to a week interacting with their supervisors at UWC where possible. In addition, students' access to electronic databases and other resources has improved exponentially as the UWC's library facilities have improved, and as the variety of information on the Internet grows.

At a support level, recognizing the differing demands of students' prior qualifications, pedagogical support strategies were put in place to equip students with the academic competences required for the more sociological orientation of public health. These included a substantial academic skills component integrated into the learning materials; more stringent candidate selection measures that included the assessment of applicants' writing and reading skills; academic development sessions at the summer and winter Schools; a written handbook aimed at developing study planning capabilities, time management skills and academic reading and discourse abilities. In addition, the Postgraduate Enrolment and Throughput Programme (PET) at the university provides writing coaches for students working on mini-theses, a system that has been effective even at a distance. Finally, strategies to monitor the academic programme were put in place from early 2004.

## Evaluative indications of impact

Evaluation of the programme has been undertaken at regular intervals, but to date this has remained formative and process-oriented. Some evaluative indications of the programme's impact have been gathered, however, although more systematic impact evaluation is sorely needed. Formative evaluations have included a process of developmental testing of selected distance learning modules over the years; ongoing student feedback on the programme and materials; a qualitative evaluation in late 2003 conducted among those students studying in the Eastern Cape, and more recently, an assessment of student progress across the years. In addition, external evaluations have been undertaken by the South African Institute for Distance Education (SAIDE) and a specialist public health evaluator, Professor Carl Taylor [Unpublished report: Welch T, Mays T: *A Distance Education Evaluation of the Postgraduate Programme in Public Health offered by the School of Public Health, UWC*; Johannesburg: SAIDE; 2006; Unpublished report: *External Evaluation by Carl Taylor, MD, Dr. PH, FRCP (Canada), Professor Emeritus Johns Hopkins School of Public Health*. UWC: SOPH; March 2006].

Evidence exists of the developmental role that the programme has played for health professionals who have engaged in it. For example, a feature that was repeatedly raised in a formative qualitative evaluation of South African students' experiences of the programme up to 2003 [Unpublished report: Alexander L: *Report of an Evaluation of Eastern Cape Students' Experiences of the SOPH Postgraduate Programme in Public Health*, *University of the Western Cape 2000–2003*. UWC: SOPH; 2006)] was a feeling of "personal empowerment". For a Provincial Programme Manager in South Africa, empowerment seemed to derive from increased knowledge: "... public health gives you that confidence ... I was a shy person, I couldn't face a crowd. It's because of the knowledge I think I am a better person ..." [from the same report].

One of the advantages of offering distance learning has been to facilitate the simultaneous integration of new conceptual knowledge with practice, through assignments and projects in which students apply new approaches to familiar problems. Students like "Thobile", a postgraduate certificate student, reported evidence of her application of new skills, for example, identifying changes in her own practice that she attributed to her recent studies. She offered this example:

"... a child presents with a physical problem, e.g. underweight, whereas often the underlying cause is a social problem at home. You counsel the mother and find that there is a lack of the social grant. ... [The Programme] changed me from the way I was seeing things ..." [from the unpublished report by Alexander, cited above].

Another student, "Nora", attributed her ability to use data for management to the basic epidemiology module, noting that:

"If a manager doesn't know how to utilise that data ... then you won't be able to monitor what you've done. You won't be able to identify the gaps. ... Because in [my area], I've got a very poor coverage throughout, I didn't even know what was lacking. Now ... I need to go there and look at my targets and then make a chart to see which areas have the largest populations. Then my graph goes like that and I say: I need to go directly there – not go running around all over the province ... So that is what I like most about it" [from the unpublished report by Alexander, cited above].

From the same study, a manager of a rehabilitation hospital, "Joe", noted how he had found immediate application of the strategic management and planning skills in his place of work, where he was responsible for 17 staff members. "Nora" was also very enthusiastic about her improved competence and understanding of her role as a new field manager.

"As a manager, you've got to have management skills and having gone through [the Programme], that has changed my working patterns. ... I was bragging about the course only yesterday because previously, I couldn't integrate my data. It's very easy now for me to monitor what I'm doing because I'm working as a manager, as a trainer, as a capacity builder – so now that I'm doing this programme, I'm able to ... look at [the] ... data to see where there are gaps and go directly to the area where there's a problem. So it's very useful ... it's making me very proud as a manager – and I'm confident!" [from the unpublished report by Alexander, cited above].

These testimonies from students have confirmed that much of the curriculum holds relevance for health workers in their day-to-day working environments.

**Figure 2:**
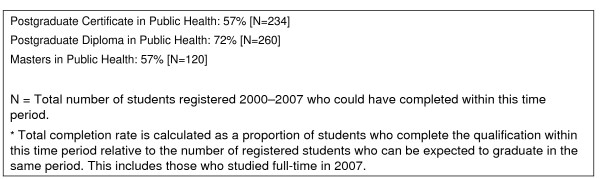
Total completion rates*, 2000–2007.

Crucial to developing distance education programmes is clarity regarding the delivering institution's objectives [9]: in our case, although the costs to both students and the institution are considerations, the primary objective has been to broaden access to public health education. The SOPH's increased intake and success rate since 2000 suggests that this is being achieved. Since commencement of the post-2000 distance education model, SOPH's reach in Africa has expanded dramatically, with almost 300 registered students in 2007 from 19 countries. Completion rates measured over seven years have improved dramatically as systems problems have been ironed out and SOPH's experience base grows (Figure 2).

These improvements must in part be attributed to selected strategies put in place over the past five years:

• creation of a comfortable learning environment by providing as much information as is manageable about the programme upfront, including learning target dates, academic strategies and time management skills;

• increased regularity of supportive communication between administrative and academic staff and students, with an emphasis on motivational communication, e.g. through regular "student memos";

• embedding academic support within assignments, which includes the requirement to perform to particular standards, support in doing so and penalties where this is not done;

• embedding in-text tasks and assignments in students' own workplace practice;

• recognizing the supportive role of assignments and striving to maintain turnaround of two to three weeks, as well as substantial quality feedback through assignments.

Another impact is making postgraduate education financially accessible. It is noteworthy that residential programmes in public health locally and overseas can only cater for a tiny minority of developing-country learners because of their costs [7]. In line with the UWC's policy on fees and access, SOPH's study fees have been kept as low as possible, amounting to approximately USD 1700 for the whole three-year qualification, which is low when compared to other institutions in South Africa and elsewhere in Africa. (This is the cost provided the student completes the MPH within the required time period of three years.) It must be noted, however, that in addition, students incur additional costs for items such as: courier delivery of their materials where the postal system is unreliable; the purchase of selected publications; their own communication and information technology expenses; and the often very high travel insurance and accommodation costs for attending contact sessions, when they can afford it.

A further criterion for judging success is containing student drop-out, which is frequently noted as very high in distance settings [10]. This often arises from "... students' sense of isolation when they study without peer or instructor interaction, insufficient self-discipline, loss of interest or discouragement owing to the slow feedback they receive in the form of graded assignments" [10]. Competition from family and workplace commitments is also cited. All these factors have affected SOPH students, but drop-out has remained low, affected by one additional factor: lack of access to information technology and academic resources, particularly in South Africa.

Other evidence of impact has been identified through two external evaluations: in 2006, Professor Carl Taylor assessed the curriculum positively, noting the following qualities: its strong focus on prevention and health promotion, which fits the need for traditional health services to move beyond clinical care; that it targets new mid-level managers who most need public health training; its flexibility allowing students to work and support their families while undertaking their postgraduate studies; its affordability to students; the combination with short intensive courses on campus without further fees; and the synergy between learning and working, which "becomes learning by doing" [Unpublished report: *External Evaluation by Carl Taylor, MD, Dr. PH, FRCP (Canada), Professor Emeritus Johns Hopkins School of Public Health*. UWC: SOPH; March 2006].

In the other external evaluation by the South African Institute for Distance Education (SAIDE), the following observation was made:

"The SOPH postgraduate programme is offering high quality distance education to a range of students who would not otherwise be able to access postgraduate studies in public health. ..."

"reviews of selected modules revealed that materials are developed according to a template that encourages carefully sequenced, interactive learning that integrates assessment requirements, in-text tasks, and readings to assist learners to achieve the outcomes of the module as a whole as well as the outcomes of individual units. In summary, they can be regarded as quality materials." [Unpublished report: Welch T, Mays T: *A Distance Education Evaluation of the Postgraduate Programme in Public Health offered by the School of Public Health, UWC*; Johannesburg: SAIDE; 2006]

## Challenges, future directions and strategies for scaling up

Despite these successes, the SOPH faces a number of challenges in delivering public health education. First, the expansion of the student body places significant strain on both the existing administrative capacity and student support systems, including tutor-markers, which need more coordination, training and moderation of their work. In particular, the workload involved in supervising mini-theses has increased considerably over the years. Our observation has been that supervising research in distance learning calls for potentially more intensive input from supervisors. This arises almost innately from the challenge of separation between the student and supervisor in distance education, making feedback more time-consuming.

Second, notwithstanding efforts to keep costs low, the cost of postage or courier delivery has escalated in recent years with the expansion of the programme to students in more geographically dispersed areas. Coupled with currency exchange rate fluctuations, the implication is that the cost of study to students from some countries such as Malawi and Zimbabwe effectively excludes some of them. This is the case as more than 70% of students registered in our programme in 2007 were funded by self or family.

Third, there have been the well-documented organizational challenges to the delivery of a distance learning programme in a university originally structured around contact and residential training [9]. The demands of the distance learner are unique, as is the learning environment required. It is often difficult to align administrative systems, and to some extent teaching and learning activities, with the time and sequence of face-to-face university programmes, consequently demanding alternative systems and structures.

Finally, academic dishonesty in the form of contracting someone else to substitute for the student in writing academic tasks is also a concern, as with any distance education or residential programme. Without getting to know students and their capabilities well, there is the possibility that students may be fraudulently aided by a third party. There has been no obvious reason to infer this so far, but it has been recognized that systems need to be strengthened to prevent this from occurring.

In order to meet the above challenges, a number of strategies are being explored, including:

• strengthening partnerships with more public health training and research institutions to increase the pool of teaching, supervising and mentoring assistance for students. Such partnerships could further provide access platforms of Internet connectivity and settings for the conduct of more regulated assessment exercises such as formal examinations;

• increasing the number of scholarships available for students. A challenge here is the demand for "full-time study" by most funding bodies in order to qualify for assistance. Further interaction with employer bodies could be undertaken to facilitate support of the adjunct expenses of studying at a distance;

• further exploration of the e-learning medium, as access to the relevant technology improves.

## Conclusion

While recognizing the many challenges faced in delivering this programme, our external and student evaluations and the growing demand for the programme suggest that public health education at UWC is meeting some of the perceived needs of health service practitioners and managers, through a teaching mode that matches their personal, economic and academic needs. Our experience suggests that remaining responsive to students' study contexts is an essential element for success of public health education programmes.

In the context of the crisis of human resources for health in Africa, training programmes of health professionals in crucial public health roles must not disrupt the provision of health services. Distance education, such as that offered at the SOPH, has the potential to do this.

## Competing interests

The authors all work for the School of Public Health, University of the Western Cape.

## Authors' contributions

LA, EI and DS were involved in the conceptualization, initial drafts and final write-up of the paper.
